# ASTHMA IN OLDER ADULTS: IDENTIFYING PHENOTYPES AND FACTORS IMPACTING OUTCOMES

**DOI:** 10.1093/geroni/igz038.045

**Published:** 2019-11-08

**Authors:** Rodney Folz

**Affiliations:** University Hospitals Cleveland Medical Center, Cleveland, Ohio, United States

## Abstract

Asthma, of all chronic diseases, has the highest disease burden attributed to environmental exposures. Few studies have attempted to characterize the prevalence of co-existing auto-inflammatory disease and asthma, or to link environmental exposure as a factor that may increase asthmatic lung obstruction and racial disparity with auto-inflammatory comorbidity. While there is an increased risk for asthma development and severity linked to certain autoimmune diseases, there is a known racial disparity in the prevalence of these autoimmune diseases. Racial and ethnic differences in the link between environmental exposures and auto-immune comorbid asthma as a potential common trigger of inflammation is not well understood. This talk will focus on developing a model to longitudinally predict asthma control and quality of life associated with home environmental triggers and volatile organic chemical (VOC) exposure in older adults and investigate the direct and indirect effect of autoimmune disease in racial disparities of the longitudinal relationships of home environmental asthma triggers on airway obstruction and functional status in older adults with asthma.

